# Sink Status and Photosynthetic Rate of the Leaflet Galls Induced by *Bystracoccus mataybae* (Eriococcidae) on *Matayba guianensis* (Sapindaceae)

**DOI:** 10.3389/fpls.2017.01249

**Published:** 2017-07-24

**Authors:** Denis C. Oliveira, Ana Silvia F. P. Moreira, Rosy M. S. Isaias, Vitor Martini, Uiara C. Rezende

**Affiliations:** ^1^Laboratório de Anatomia e Desenvolvimento Vegetal, Instituto de Biologia, Universidade Federal de Uberlândia Minas Gerais, Brazil; ^2^Laboratório de Anatomia Vegetal, Departamento de Botânica, Instituto de Ciências Biológicas, Universidade Federal de Minas Gerais Minas Gerais, Brazil

**Keywords:** biotic stress, ROS, photochemistry, stress dissipation, plant galls, galling insects

## Abstract

The galling insect *Bystracoccus mataybae* (Eriococcidae) induces green and intralaminar galls on leaflets of *Matayba guianensis* (Sapindaceae), and promotes a high oxidative stress in host plant tissues. This biotic stress is assumed by the histochemical detection of hydrogen peroxide, a reactive oxygen species (ROS), whose production alters gall physiology. Thus, we hypothesize that high levels of nutrients are accumulated during gall development in response to a local maintenance of photosynthesis and to the galling insect activity. Moreover, the maintenance of low levels of photosynthesis may guarantee O_2_ production and CO_2_ consumption, as well as may avoid hypoxia and hypercarbia in gall tissues. To access the photosynthesis performance, the distribution of chlorophyllous tissues and the photochemical and carboxylation rates in gall tissues were analyzed. In addition, histochemical tests for hydrogen peroxide and phenolic derivatives were performed to confirm the biotic stress, and set the possible sites where stress dissipation occurs. The contents of sugars and nitrogen were evaluated to quantify the gall sink. Currently, we assume that the homeostasis in gall tissues is ruptured by the oxidative stress promoted by the galling insect activity. Thus, to supply the demands of gall metabolism, the levels of water-soluble polysaccharides and starch increase in gall tissues. The low values of maximum quantum efficiency of PSII (*F*_v_/*F*_m_) indicate a low photosynthetic performance in gall tissues. In addition, the decrease of PSII operating efficiency, (*F*’m–*F*’)/*F*’m, and Rfd (instantaneous fluorescence decline ratio in light, to measure tissue vitality) demonstrate that the tissues of *B. mataybae* galls are more susceptible to damage caused by stressors than the non-galled tissues. Thus, the high oxidative stress in gall developmental sites is dissipated not only by the accumulation of phenolic derivatives in the protoplast, but also of lignins in the walls of neoformed sclereids.

## Introduction

As a novel approach on the discussion of the impact of galling insects on the photosynthesis of their host plant tissues, we herein address the association of photochemical activity with carboxylation rate in gall tissues. We take for granted that the integrity of the photochemical and carbon assimilatory apparatus should be maintained in green gall tissues. Moreover, a high oxidative stress imposed by the galling herbivores in host plant tissues (Oliveira et al., 2011) should rupture the homeostasis in gall developmental sites (Isaias et al., 2015), and demand metabolic reactions, such as the overproduction of phenolic derivatives, and the establishment of a sink of photoassimilates.

The detrimental effects of galling insects on the performance of their host plants are linked to the establishment of sinks in gall sites (reviewed by Fernandes, 1987; Fernandes and Santos, 2014). These sinks drain nutrients from other plant parts (Mani, 1964; Castro et al., 2012; Huang et al., 2014), which is especially true for galls induced by sucking insects. The feeding activity of these insects in specific plant tissues (e.g., the phloem) affects the carbon-partitioning mechanisms within the host plant compartments, and alters the balance among source and sink tissues. Changes in source-sink relationships can reduce the photosynthetic capacity in remaining non-galled leaf tissues around the gall developmental sites (Zangerl et al., 2002; Nabity et al., 2009). Consequently, galls often reduce the development and performance of their host plants, leading to a reduction in flower, fruit, seed, and biomass production, the main purpose of plant photosynthesis (e.g., McCrea et al., 1985; Sacchi et al., 1988; Fernandes et al., 1993).

Galls induced by Cecidomyiidae (Diptera) can cause a decrease in photosynthetic rates (Andersen and Mizell, 1987; Larson, 1998; Florentine et al., 2005; Huang et al., 2014), and function as strong sinks of photoassimilates from their host leaves. This pattern was observed in the horn-shaped gall induced by a Cecidomyiidae on *Copaifera langsdorffii* (Castro et al., 2012), and in the ovoid and obovate galls induced by *Daphnephila taiwanensis* and *D. sueyenae* (Cecidomyiidae) on *Machilus thumbergii* (Huang et al., 2014). Nevertheless, some galls do not cause significant changes in the photosynthesis of their host organs, as the galls induced by the aphid *Melaphis rhois* on *Rhus glabra* (Larson, 1998), and by the psyllid *Pseudophacopteron aspidospermi* on *Aspidosperma australe* (Oliveira et al., 2011; Malenovský et al., 2015). Other galls may be even beneficial to their host plants, as those induced by cynipid wasps in *Acacia pynantha*, whose photosynthetic rates are higher than those of similarly-age non-galled leaves (Dorchin et al., 2006).

The common traits of galls with neutral or positive photosynthetic capacity seem to be the intralaminar position and/or the green color. Green galls may have the potential to photosynthesize, primarily, by maintaining the structure of their chlorophyll tissues and photochemical apparatus (Oliveira et al., 2011). Consequently, in gall developmental sites, oxygen production and CO_2_ consumption can avoid hypoxia and hypercarbia (Castro et al., 2012; Isaias et al., 2015), which may be related to tissue compactness, both in non-galled plant compact tissues (Moreira et al., 2009) and in gall parenchyma. In addition, the photochemical activity minimizes the triplet chlorophyll (^^∗^^Chl^3^) production, which ends up in the formation of ROS molecules (Pavlovic, 2012). Some of these molecules, such as the H_2_O_2_, have been histochemically detected in galls, and are indicative of a high oxidative stress imposed by the galling insects (Oliveira et al., 2011, 2014a, 2016; Isaias et al., 2015).

The galls induced by *Bystracoccus mataybae* (Eriococcidae) on the leaflets of *Matayba guianensis* (Sapindaceae) (Hodgson et al., 2013), the current model of study, fits the intralaminar position and are green, requisites for the maintenance of the photosynthetic activity in their tissues. We herein use this host plant-galling insect system to analyze the counterbalance between the photosynthetic activity and the indicatives of high oxidative stress in gall developmental site. Moreover, we unite a set of analyses, previously test for other host plant-galling insect systems, and stepped forward with new approaches on photosynthesis to reveal its contribution for homeostasis in gall tissues.

## Materials and Methods

### Study Area and Plant–Insect System

**FIGURE 1:**
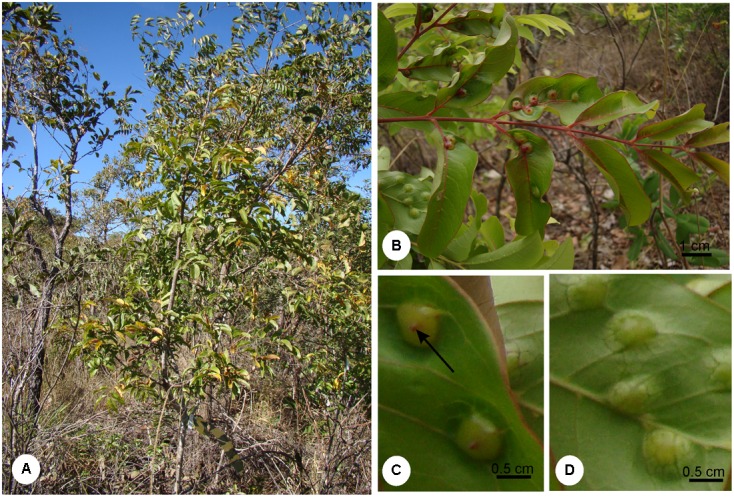
**(A)** Shrub of *Matayba guianensis* (Sapindaceae) in a population located at Estação Ecológia do Panga, municipality of Uberlândia, Minas Gerais, Brazil. **(B)** Galled leaf. **(C)** Detail of a leaflet adaxial surface evidencing gall ostiole (arrow). **(D)** Detail of the leaflet abaxial surface evidencing gall protuberance.

*Matayba guianensis* (Sapindaceae) has a wide distribution in Brazilian Cerrado areas with oligotrophic soils (Ratter et al., 1996). It is a shrub (**Figure 1A**), 2 m high on average, with galls induced by *B. mataybae* (Eriococcidae) (Hodgson et al., 2013) on its leaflets. The galls are intrallaminar (Isaias et al., 2013) and induced by the galling insect only on the abaxial surface of the leaflets, but protrude both to the adaxial (**Figure 1B**) and to the abaxial leaflet surface (**Figures 1C,D**). They are parenchymatic, and the chlorophyllous tissues occupy the major portion of gall structure. There are two interconnected nymphal chambers, which shelters one individual of *B. mataybae*, and no parasitoids. For physiological and histochemical analyses, mature galls with adult females (Hodgson et al., 2013) and non-galled mature leaves were sampled in a population of *M*. *guianensis* located in an area of 403.85 ha of Cerrado *sensu stricto* at Estação Ecológica do Panga (19° 10′S, 48°24′O) in Uberlândia municipality, Minas Gerais state, Brazil, during the wet season. The wet season ranges from October to March with around 1,550 mm of annual precipitation and 22°C of annual temperature (Cardoso et al., 2009).

### Relative Water Content (RWC), Specific Mass, and Succulence

The relative water content (RWC), specific mass, and suculence were measured in disks (8 mm in diameter) of the middle portion of non-galled leaflets (*n* = 30) and of mature galls (*n* = 40) from six individuals, at 7:00 am. The RWC was obtained from the formula (FM-DM)/(TM-DM) ×100, where FM was the fresh mass, DM the dry mass, and TM the turgid mass (Turner, 1981). The specific mass was obtained from the formula DM/A, where A is the known area (Witkowski and Lamont, 1991), and succulence was obtained from the formula: SU = (TM-DM)/DM (Ogburn and Edwards, 2012).

### Gas Exchanges, Stomatal Density, and Intercellular Spaces

Carbon assimilation (*A*), internal concentration of carbon (*ci*), evapotranspiration (*E*), and stomatal conductance (*g*) were evaluated in non-galled leaflets (*n* = 10) and mature galls (*n* = 10), at 8:00 am, using the Infrared Gas Analyzer-IRGA (Infra-Red Gas Analyser, LCA-Pro, Analytical Development Co. Ltd., England). For the measurement of the gas exchange, petrolatum (vaseline) was applied around the galls to eliminate the non-galled leaf surface areas that should interfere in the analysis (Duarte et al., 2005). Mathematical correction was made for standardization of the areas, with the results expressed per cm^2^, and taking into account that the mature gall mean area was 1.48 cm^2^. Stomatal density was measured in epidermal fragments of the abaxial surface of galls and non-galled leaflets (0.5 cm^2^) detached by immersion in 50% hypochlorite, during 2 days (Kraus and Arduin, 1997), washed in water, and stained with 0.5% ethanolic safranin (Johansen, 1940). The fragments were mounted in Kaiser’s jelly glycerin (Kraus and Arduin, 1997). The number of stomata per area (mm^2^) was counted with the aid of a drawing tube coupled to a light microscope (Olympus Optical Co. Ltd, CH30 RF100). Intercellular spaces were measured using the samples of non-galled leaf (*n* = 4) and galls (*n* = 10) fixed in FAA (Kraus and Arduin, 1997), dehydrated in ethanol series, embedded in 2-hydroxyethyl methacrylate (Historesin, Leica^®^ Instruments, Germany), and sectioned in a rotary microtome (4–5 μm). The sections were stained with toluidine blue at pH 4.0 (O’Brien and McCully, 1981) and mounted with Entellan^®^. For each sample, five sections were measured and analyzed in the software of the optical microscope Leica^®^ DM500 coupled with HD5000 camera.

### Sugar and Nitrogen Contents

The content of nutrients (nitrogen and carbohydrates) were evaluated in non-galled mature/intact leaflets and in galls (*n* = 10, from 10 individuals). For carbohydrate determination, samples of non-galled and galled leaflets were treated in a microwave oven for 30 s (Marur and Sodek, 1995), dried at 50°C for 48 h, and ground in a mortar. The extraction of total soluble sugars (TSS) was performed in a solution of methanol: chloroform: water (12:5:3 v/v) (Bielski and Turner, 1966). The content of water-soluble polysaccharides (WSP) was obtained by the residue resuspension in 10% ethanol (Shannon, 1968). A new extract obtained from the pellet resuspension in 30% perchloric acid (McCready et al., 1950) was taken to measure the content of starch. The dosage was performed in triplicate by colorimetric analysis using the phenol-sulfuric acid method (Dubois et al., 1956), modified by Chow and Landhäusser (2004), using glucose as standard.

The nitrogen content was obtained according the Kjeldahl method (Tedesco et al., 1995). Plant material was dried in an oven of forced circulation at 50°C for 48 h. The samples of non-galled and galled leaflets were ground in a mortar, and 250 mg of dry mass of each sample were submitted to the sulfuric digestion. Later on, the material was submitted to steam distillation (distiller Tecnal TE-0363, Brazil), where the ammonia released in the form of NH_4_OH was trapped in 2% boric acid, and titrated against standardized 0.02 N hydrochloric acid.

### Photosynthetic Pigment Contents and Chlorophyll Fluorescence Measurements

Fresh non-galled leaflets (*n* = 30) and galls (*n* = 30) of six individuals were cut in disks of 0.8 cm^2^, weighed, and immersed in 80% acetone (v:v) for the extraction of pigments. The extracts were analyzed in a spectrophotometer, and the contents of chlorophyll and carotenoids were calculated following the equations proposed by Lichtenthaler and Wellburn (1983).

Fluorescence quenching analysis was performed in non-galled and galled leaflets (*n* = 5) (at 8:00 am) using a modulated fluorescence imaging apparatus, Handy Fluorcam PSI (Photo Systems Instrument, Czech Republic). The maintenance of the integrity of the electron chains and the capacity of non-galled and galled tissues for photosynthesizing were demonstrated after a dark-adapted (30 minutes) time, and exposition to various light treatments following the software protocol – Quenching^1^ (Photo Systems Instruments, Version 2). The following parameters were used in this study: *F*_0_ (minimum fluorescence of PSII in dark-adapted state); Fm (maximum fluorescence of PSII in dark-adapted state); *F*v/*F*m (maximum PSII quantum yield in dark-adapted state, where *F*v = *F*m–*F*_0_); (*F*′m–*F*′)/*F*′m (PSII operating efficiency, where *F*′m is the fluorescence signal when all PSII centers are closed in the light-adapted state and F′ is the measurement of the light-adapted fluorescence signal); Rfd (instantaneous fluorescence decline ratio in light); NPQ_DN_ (instantaneous non-photochemical quenching during dark relaxation) and NPQ_Lss_ (steady-state non-photochemical quenching) (Genty et al., 1989; Oxborough, 2004).

### Histochemical Analysis

Handmade sections of fresh mature galls were used for the detection of hydrogen peroxide, a reactive oxygen species (ROS), phenolic compounds, proanthocyanidins (flavonoids), and lignins. This ROS was detected with DAB (3,3′-diaminobenzidin) for 15 min in the dark and compared to blank sections (Rosseti and Bonatti, 2001; Oliveira and Isaias, 2010a; Oliveira et al., 2011; Isaias et al., 2015). For the detection of phenolic compounds, the samples were incubated in 2% ferrous sulfate in 10% formalin for 10 min, and then washed in water (Gahan, 1984); proanthocyanidins were detected after fixation in 2% caffeine–sodium benzoate for 5 min, followed by incubation in DMACA (*p*-dimethylaminocinnamaldehyde) for 2 h (Feucht et al., 1986). In addition, blank sections (without staining) were used to check chlorophyllous tissue distribution. Lignins were detected by autofluorescence with a DAPI filter (Chomicki et al., 2014) and by Wiesner reagent (2% phloroglucinol in 1N HCl) (Johansen, 1940).

### Statistical Analysis

The data were submitted to ANOVA, Shapiro–Wilk normality test, and comparison of means by Tukey test for parametric and Wilcoxon test for non-parametric analyses, respectively, on JMP^®^5.0 Software (Sas Institute Inc., NC, United States, 2002).

## Results

### Carbohydrate, Nitrogen, and Water Content

**FIGURE 2:**
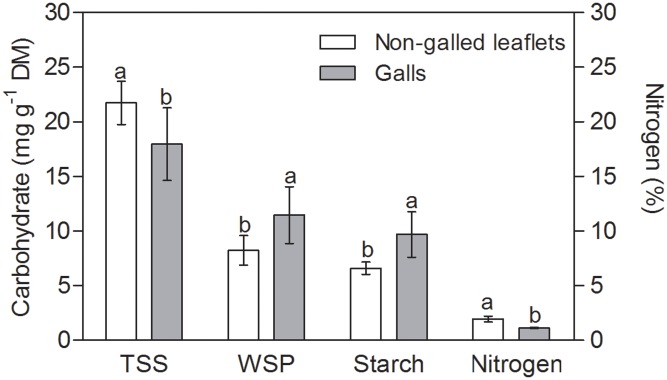
Sugar and nitrogen contents in non-galled leaflets of *Matayba guianensis* and in galls induced by *Bystracoccus mataybae* (means ± standard deviation). TSS, total soluble sugars; WSP, water soluble polyssacharids. Values followed by the same letters, for each substance, do not differ significantly at *P* ≤ 0.05 level by the Tukey’s test.

The TSS content was 20% higher in non-galled tissues than in mature galls (**Figure 2**). WSP and starch contents were 30 and 25% higher in galls than in non-galled tissues, respectively. The nitrogen content was 15% higher in non-galled tissues than in galls (**Figure 2**), and the RWC was higher in mature galls than in non-galled tissues (**Table 1**). Succulence and specific mass were 3-times higher in galls than in non-galled tissues (**Table 1**).

**Table 1:** Means ± standard deviation (*n* = 10) for physiological characteristics related to water content and physiological profile of non-galled tissues and galls induced by *Bystracoccus mataybae* on *Matayba guianensis*.

	Galls	Non-galled tissues	*P*
Stomatal density (n_st_ mm^-2^)	23.0 ± 22.59^b^	678.5 ± 131.3^a^	<0.0001
Stomatal conductance (mol m^-2^ s^-1^)	0.035 ± 0.040^b^	0.112 ± 0.024^a^	*<0.001*
Internal concentration of carbon (μmol mol^-1^)	308.15 ± 91.76^a^	314.11 ± 81.36^a^	*0.07*
Carbon assimilation (μmol_CO2_ m^-2^s ^-1^)	0.81 ± 1.79^b^	2.72 ± 0.57^a^	*<0.0001*
Evapotranspiration (E)	0.43 ± 2.98^b^	0.78 ± 0.11^a^	*0.04*
Relative water content (RWC) (%)	96.88 ± 37.4^a^	67.78 ± 38.6^b^	*<0.001*
Specific mass (g_DM_ cm^-2^)	88.6 ± 23.3^a^	51.3 ± 13.3^b^	*<0.0001*
Succulence (g_H2O_ g^-1^_FM_)	260.34 ± 72.8^a^	75.5 ± 20.6^b^	*<0.0001*
Intercellular spaces (mm^2^ mm^-2^_leaf_)	6.7 × 10^-3^ ± 8.3 × 10^-3b^	63.9 × 10^-3^ ± 12 × 10^-3a^	*<0.001*

*Values followed by the different letters differ significantly by the Tukey’s test at 5% of probability*.

### Stomatal Density, Gas Exchanges, and Intercellular Spaces

The stomatal density was higher in the abaxial than in the adaxial leaflet epidermal surface, and thus, the leaflets were herein considered as functionally hypostomatic with anysocitic stomata. Furthermore, the stomatal density was higher in leaflets than in galls (**Table 1**). The stomatal conductance (*g*) and carbon assimilation (A) were 3-times higher in leaflets than in galls. The internal carbon concentration (*ci*) was similar in leaves and galls, even though galls had greater amplitude of data (**Table 1**). The intercellular spaces in gall tissues decrease significantly when compared to non-galled leaf (**Table 1** and Supplementary Figure 1).

### Pigment Content and Chlorophyll *a* Fluorescence

Total chlorophyll and carotenoid contents were 4-times higher in non-galled leaflets. In addition, the ratio of chlorophyll *a*/*b* and chlorophyll/carotenoids were slightly higher in non-galled leaflets than in galled tissues (**Table 2**).

**Table 2:** Photosynthetic pigment content (means ± standard deviation, *n* = 30) in *Matayba guianensis* leaflets and galls induced by *Bystracoccus mataybae*.

	Chl (*a+b*) (mg g^-1^ FM)	Carotenoids (mg g^-1^ FM)	Chl a/b	Chl (*a+b*)/carot
Non-galled tissues	1.65 ± 0.3^^a^^	0.30 ± 0.05^^a^^	3.25 ± 0.6^^a^^	5.56 ± 0.9^^a^^
Galls	0.39 ± 0.09^^b^^	0.08 ± 0.01^^b^^	2.60 ± 0.2^^b^^	4.96 ± 0.4^^b^^
*P*	*<0.0001*	*<0.0001*	*<0.0001*	*<0.05*

*Values followed by the different letters differ significantly by the Tukey’s test at 5% of probability*.

**FIGURE 3:**
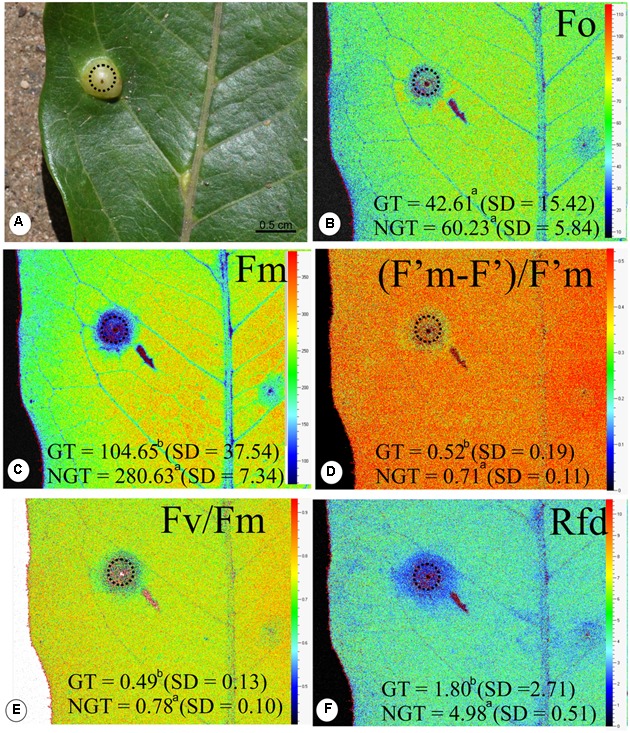
Fluorescence quenching analysis performed (means ± standard deviation) in non-galled tissues of *Matayba guianensis* (Sapindaceae) and in galls induced by *Bystracoccus mataybae* (Eriococcidae) using a modulated fluorescence imaging apparatus (Handy Fluorcam PSI – Photo Systems Instrument, Czech Republic). **(A)** Leaflet with a gall (doted circle). **(B)** Imaging of F_0_ (minimum fluorescence of PSII in dark-adapted state). **(C)** Imaging of Fm (maximum fluorescence of PSII in dark-adapted state). **(D)** Imaging of (*F*’m–*F*’)/*F*’m (PSII operating efficiency). **(E)** Imaging of *F*v/*F*m (maximum PSII quantum yield in dark-adapted state). **(F)** Imaging of Rfd (instantaneous fluorescence decline ratio in light). GT, galled tissue; NGT, non-galled tissue, SD, standard deviation. Values followed by the different letters differ significantly by the Wilcoxon test.

**FIGURE 4:**
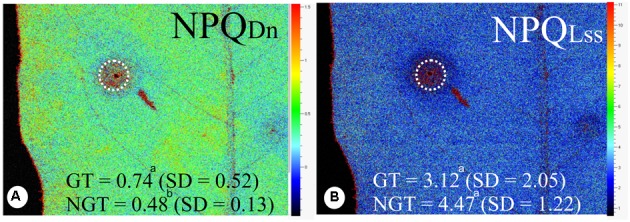
Fluorescence quenching analysis performed in non-galled tissues of *Matayba guianensis* (Sapindaceae) and in galls induced by *Bystracoccus mataybae* (Eriococcidae) using a modulated fluorescence imaging apparatus (Handy Fluorcam PSI – Photo Systems Instrument, Czech Republic). **(A)** Imaging of NPQ_DN_ (instantaneous non-photochemical quenching during dark relaxation). **(B)** Imaging of NPQ_Lss_ (steady-state non-photochemical quenching). GT, galled tissue; NGT, non-galled tissue; SD, standard deviation. Values followed by the different letters differ significantly by the Wilcoxon test.

There is no difference in the minimum fluorescence of PSII in dark-adapted state (F_0_) between galled and non-galled tissues, and the maximum fluorescence of PSII in dark-adapted state (Fm) was 130% higher in the non-galled than in galled tissues (**Figures 3A–C**). The (*F*’m–*F*’)/*F*’m, *F*v*/F*m, and *Rfd* values were higher in non-galled than in galled tissues (**Figures 3D–F**). The instantaneous non-photochemical quenching during dark relaxation – NPQ_Dn_ is higher in galled tissues than in non-galled tissues, while steady-state non-photochemical quenching in light – NPQ_Lss_ do not show statistical difference (**Figures 4A,B**).

### Histochemical Analysis

**FIGURE 5:**
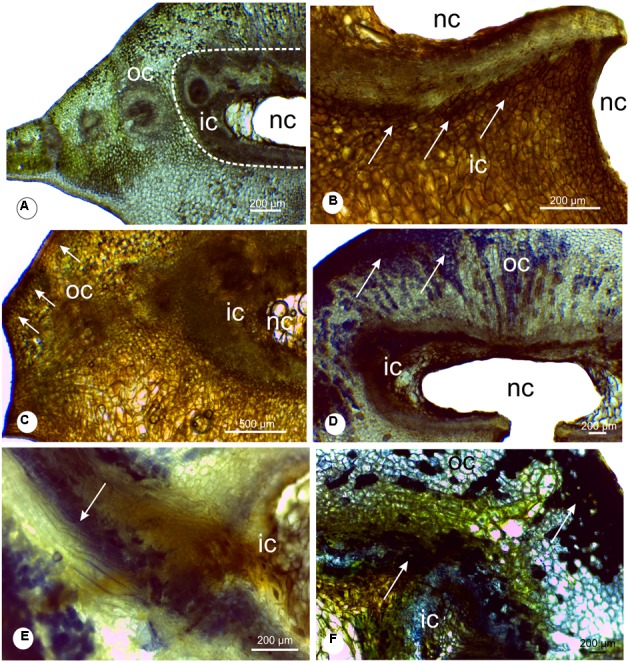
Histochemical analysis of leaflet galls induced by *Bystracoccus mataybae* (Eriococcidae) on *Matayba guianensis* (Sapindaceae). **(A)** Transverse section of a gall with no staining, evidencing the distribution of the chlorophyllous tissue. **(B)** Histochemical detection of oxygen reactive species (ROS) with DAB (3,3′-diaminobenzidine). The positive reaction is evidenced by the brown spots. **(C)** Histochemical detection of ROS with DAB in the outer and inner cortex of galls. **(D)** Proanthocyanidins were detected with DMACA (*p*-dimethylaminocinnamaldehyde) at the chlorophyllous tissue. **(E)** Detail of proanthocyanidins in cells around the nymphal chamber, and in lignified cells. **(F)** Phenolic compounds detected with 2% ferrous sulfate in 10% formalin in gall outer cortical tissues, continuous to the chlorophyllous tissue. ic, inner cortex; nc, nymphal chamber; oc, outer cortex;

**FIGURE 6:**
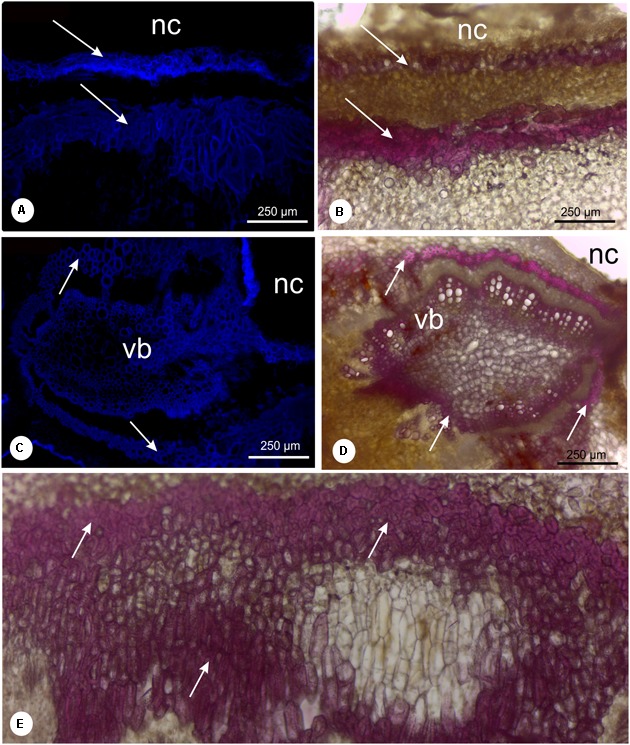
Lignin detection in leaflet galls induced by *Bystracoccus mataybae* (Eriococcidae) on *Matayba guiananeis* (Sapindaceae) by fluorescence method with DAPI filter **(A,C)** and histochemical test with 2% phloroglucinol-HCL **(B,D,E)**. **(A,B)** Detail of the ostiole on the abaxial surface of a gall with lignified cells (arrow). **(C,D)** Vascular bundle adjacent to the nymphal chamber surrounded by fibers (arrows). **(E)** Intense lignification around the nymphal chamber. nc, nymphal chamber; os, ostiole; vb, vascular bundles.

The chlorophyllous tissue occurs specially in the outer cortex, displayed in green color (**Figure 5A**). The hydrogen peroxide molecules were detected in the chlorophyllous tissue and in the cells around the chamber (**Figures 5B,C**). Proanthocyanidins (**Figures 5D–E**) and phenolics (**Figure 5F**) were detected mainly in the cells of the gall outer cortex, co-occurring with the ROS (**Figures 5D–F**). The analysis with DAPI filter and Wiesner reagent demonstrated lignified cells in light blue and red coloration around the nymphal chamber (**Figures 6A,B,E**) and in the vascular bundles associated to the nymphal chamber (**Figures 6C,D**).

## Discussion

The intralaminar and green status of the *Bystracoccus mataybae* galls on *Matayba guianensis* should guarantee the integrity of the photosynthetic activity. Nevertheless, it is impaired by the high oxidative stress imposed by the galling insect respiration and feeding inside the larval chamber. Moreover, the changes in source-sink relationship in gall developmental sites (Castro et al., 2012), and the development of a compact parenchyma (Oliveira and Isaias, 2010b; Isaias et al., 2014) have also occurred and impaired the homeostasis at gall developmental site. Both the galling insect and the host plant attempt to recover the homeostasis, which involves ROS scavenging, and the production of phenolic derivatives by host plant organs (Isaias et al., 2015; Oliveira et al., 2016). In such perspective, the production of phenolics is not only involved in plant defense, but, alternatively, promotes stress dissipation in host plant-galling insect systems.

### Effects of *Bystracoccus mataybae* Activity on Host Plant Structure and Physiology

Even though *B. mataybae* directly feeds in phloem cells of *M*. *guianensis*, the functionality of other tissue compartments in gall developmental sites is also affected. The first influence regards the antagonist effect of the reduction in CO_2_ assimilation and the enhancing in the sink of nutrients in gall structure. The higher rate of CO_2_ assimilation in non-galled leaflets of *M. guianensis* relates both to the high photochemical activity and to the diffusion of gases through the spongy parenchyma in comparison to the compact cortical parenchyma of *B. mataybae* galls. This interpretation is supported by the report on galls induced by *Pemphigus betae* (Aphididae) on *Populus angustifolia* (Salicaceae) (Larson, 1998), where the compactness of gall tissues implies in the loss of photochemical activity.

The loss of intercellular spaces, parenchyma hyperplasia and cell hypertrophy in *M. guianensis* leaflet galls also confer higher succulence and RWC. The RWC and leaf succulence are strongly related to leaf thickness and to the area of water storage cells (Ogburn and Edwards, 2012). The increase of RWC in *M. guianenis* cells seems to be a necessary condition for cell growth and gall formation, which is directly related to the turgor pressure imposed by the vacuoles on cell walls (Cosgrove, 1986). In addition, the flow of photoassimilates to the sink organ (currently, the gall) also depends on water equilibrium (Thompson and Holbrook, 2003), and on the control of stomata opening and closure (Franks and Farquhar, 2007). Nevertheless, alterations in the patterns of epidermal cell differentiation usually culminate in the development of non-functional stomata (Oliveira et al., 2006; Oliveira and Isaias, 2010b; Isaias et al., 2011). The non-functional stomata form a barrier for CO_2_ influx (and efflux) in gall tissues, which together with the decreasing in stomatal density, as observed in *B. mataybae* galls, negatively affects stomata conductance and the photosynthetic rates.

The decreasing of intercellular spaces in the galls on *M. guianensis* enhances the resistance for oxygen diffusion, and favors hypoxia (*cf*. Pincebourde and Casas, 2016). Also, the consumption of molecular oxygen by the respiration of plant cells and of the galling insect ends up in CO_2_ production, which is consumed in the Calvin-Benson cycle of photosynthesis avoiding hypercarbia. In summary, even though the photosynthetic activity is not capable of supporting gall development, it is important to maintain tissue stability and the aerobic gall metabolism (Castro et al., 2012). In addition, the low gas exchanges and the decreasing of the PSII functioning is a consequence of the structural alterations not only in the dermal but also in the ground system at gall developmental site. Similarly to the galls induced by a Cecidomyiidae on *Copaifera langsdorffii* (Castro et al., 2012), the photochemical activity in galls of *B. mataybae* is not enough to supply their energy demand, and a strong sink is established. The sink and consequent high concentration of carbohydrates support not only gall development and metabolism, but also the galling insect diet (Hartley, 1998; Oliveira et al., 2006; Isaias et al., 2014). The high content of carbohydrates, mainly WSP, is a consequence of an increase in cell wall components. The components of cell wall matrix are responsible for cell shape, adhesion, mechanical properties and signaling (Willats et al., 2001), which are correlated to the structural dynamics of gall development (Formiga et al., 2013; Carneiro et al., 2014a; Oliveira et al., 2014b). In addition to cell wall dynamics, the diet of the gall-inducer (Bronner, 1992) is an additional strong driven force for the accumulation of another class of carbohydrates, the starch, which is enzymatically broken into sucrose, fructose and glucose in gall developmental sites (Oliveira and Isaias, 2010a). Accordingly, the low consumption of these energetic molecules reveals the low metabolism of mature *M. guianensis* galls.

### Photosynthetic Apparatus and Strategies to Stress Dissipation

During gall development, the disorganization of thylakoid system in the chloroplast and the presence of plastoglobules may occur; however, these structural events are not necessarily accompanied by a decrease of photosynthetic performance (Oliveira et al., 2011). The relative low levels of photosynthetic pigments in the galls of *B. mataybae* on *M. guianensis* seem to be a direct effect of hyperplasia and cell hypertrophy, common responses of plant tissues to gall induction and development (Yang et al., 2003; Oliveira et al., 2011; Dias et al., 2013). Both processes of hyperplasia and cell hypertrophy, as well as the increase in water content and succulence, cause a spatial dilution of pigment contents in area. Such antagonist developmental relationship, i.e., the reduction of photosynthetic pigments and relative enhance in tissue area, has been observed in the globoid galls induced by *Nothotrioza myrtoidis* on *Psidium myrtoides* (Carneiro et al., 2014b), and by a Cecidomyiidae on *A. spruceanum* (Oliveira et al., 2011). Also, the horn-shaped galls induced by a Cecidomyiidae on *Copaifera langsdorffii* (Castro et al., 2012), the lenticular galls induced by *Pseudophacopteron aspidospermii* on *Aspidosperma australe* (Oliveira et al., 2011; Malenovský et al., 2015), and the ovoid and obovate galls induced by *Daphnephila taiwanensis* and *D. sueyenae* on *Machilus thumbergii* (Huang et al., 2014) have reduced photosynthetic pigments. However, the alterations in pigment contents do not seem to alter gall metabolism in a convergent way. The decreasing in chlorophyll content in gall developmental sites implies in a reduction of the quantum efficiency, because chlorophyll is responsible for PAR absorption, the first step for chemical energy formation. In addition, the decrease of *F*v/*F*m, (*F*’m–*F*’)/*F*’m, and Rfd indicates that *M. guianensis* gall tissues are impacted by the stress generated by the galling insect behavior, as proposed by Lichtenthaler and Miehé (1997) for plants under different stress conditions.

In the galls of *B. mataybae* on *M. guianensis*, the NPQ_DN_ is high when compared to the non-galled tissues, while the NPQ_Lss_ is similar. The high levels of NPQ are usually associated with high levels of carotenoid content, which are responsible for the energy dissipation during the xanthophyll cycle (Demmig-Adams et al., 1996). However, the galls of *B. mataybae* maintain the same levels of NPQ_Lss_ but low levels of carotenoids when compared to non-galled tissues of *M. guianensis*. We consider that the formation of ROS may come from the energy that is neither used in the qP (photochemical quenching) nor dissipated in NPQ, in an overlapping process.

Currently, the main stressors described for gall tissues are the ROS, especially hydrogen peroxide (Oliveira et al., 2011, 2014a), which demands stress dissipation (ROS scavenging) toward tissue homeostasis (Isaias et al., 2015). The concomitant localization of hydrogen peroxide and phenolics in gall tissues (Bedetti et al., 2014; Suzuki et al., 2015) led to the interpretation of the involvement of phenolic derivatives in the reduction of the oxidative stress. Herein, we assume cell wall lignification as an additional mechanism of stress dissipation (Akhtar et al., 2010; Isaias et al., 2015) in gall microenvironment. The co-occurrence of phenolics, proanthocyanidins, and hydrogen peroxide led us to propose the relationship of these secondary metabolites with ROS scavenging in the chlorophyllous tissues of *B. mataybae* galls on *M. guianensis*. Such assumption is based on the dependence of ROS generation (hydroxyl radical) and the conversion of the monolignols, *p*-coumaryl, coniferyl, and synapil alcohols, into phenoxy-radical for the biosynthesis of some phenolic derivatives, such as the lignins (Boerjan et al., 2003). Such interdependence implies in ROS consumption and dissipation along lignin biosynthesis (Grace and Logan, 2000; Blokhina et al., 2003), whose direct consequence may be the homeostasis in gall tissues.

## Conclusion

Currently, the knowledge on gall metabolism has evolved toward the role of photosynthesis as a mechanism to solve the antagonism between the favorable green intralaminar structure and the loss of functionality of dermal and ground systems. The hypoxia and the hypercarbia, consequences of low gas diffusion and stomata density, do not impair gall development, and the homeostasis is guaranteed due to metabolism strategies, such as the maintenance of quantum efficiency. Moreover, the high oxidative stress in gall developmental sites is dissipated not only by the accumulation of phenolic derivatives in the protoplast, but also of lignins in the walls of neoformed sclereids.

## Author Contributions

DO, AM, VM, and UR, data sampling and analysis. DO, AM, VM, RI, and UR analysis and manuscript writing.

## Conflict of Interest Statement

The authors declare that the research was conducted in the absence of any commercial or financial relationships that could be construed as a potential conflict of interest.

## Footnotes

1 www.psi.cz
