# Supplementary figures and images for: Sink Status and Photosynthetic Rate of the Leaflet Galls Induced by Bystracoccus mataybae (Eriococcidae) on Matayba guianensis (Sapindaceae)
Source: Front Plant Sci. 2017 Jul 24;8:1249. doi: 10.3389/fpls.2017.01249 (PMC5522869; doi:10.3389/fpls.2017.01249)

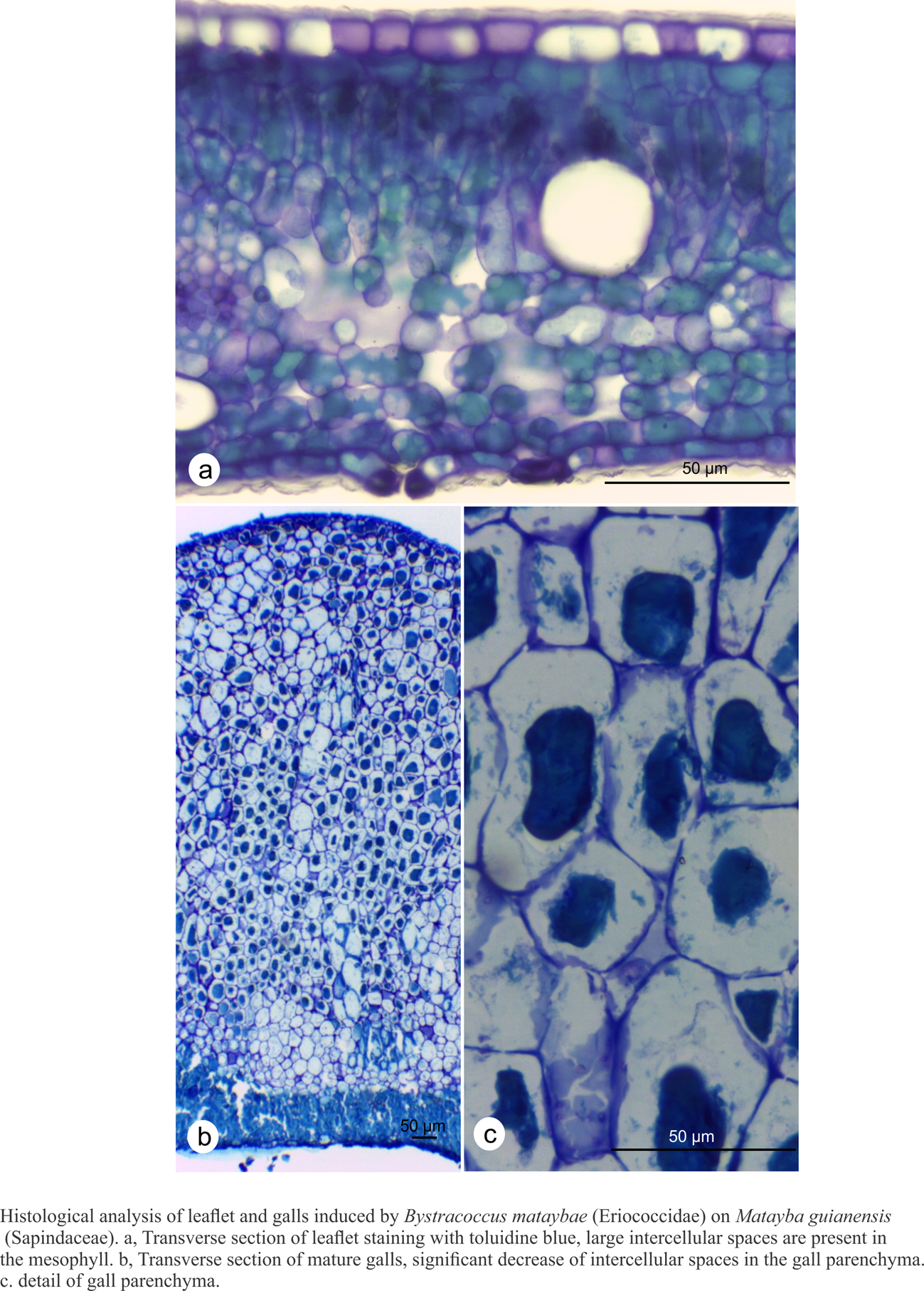

Supplement: Supplementary file 1 [file Image_1.TIF]
